# Enhancing Drug Utilization Efficiency *via* Dish-Structured Triboelectric Nanogenerator

**DOI:** 10.3389/fbioe.2022.950146

**Published:** 2022-07-06

**Authors:** Qu Chen, Wenjing Deng, Jingjin He, Li Cheng, Pei-Gen Ren, Yang Xu

**Affiliations:** ^1^ Institute of Biomedicine and Biotechnology, Shenzhen Institute of Advanced Technology, Chinese Academy of Sciences, Shenzhen, China; ^2^ Shenzhen International Institute for Biomedical Research, Shenzhen, China; ^3^ School of Basic Medical Sciences, Southern Medical University, Guangzhou, China; ^4^ School of Materials and Energy, Lanzhou University, Lanzhou, China; ^5^ Center for Energy Metabolism and Reproduction, Shenzhen Institute of Advanced Technology, Chinese Academy of Sciences, Shenzhen, China; ^6^ Shenzhen College of Advanced Technology, University of Chinese Academy of Sciences, Shenzhen, China

**Keywords:** doxorubicin, dish-structure, triboelectric nanogenerator, drug utilization, enhanced efficiency

## Abstract

Due to the finding of severe side effects and low therapeutic efficacy with cancer chemotherapy, there still remains a great challenge to benefit patients with curative effect. In this work, we designed a self-powered drug delivery system comprising a current source derived from the disk TENG (D-TENG) and a pair of Au electrodes. Thus, cells seeded within the electrode gap could be stimulated by the current followed by D-TENG`s work. Under the rotation frequency of about 7.4 Hz, the peak output current and voltage of the D-TENG reached 3.7 μA and 135 V and achieved an average of 2.8 μA of output current. Furthermore, the D-TENG also showed its good stability to output steady current in a long-term condition. When applying the electric stimulation by this self-powered drug delivery system, a chemotherapy drug, doxorubicin (DOX), had significant uptake by cancer cells. Therefore, utilizing a novel TENG device as a part of chemotherapy would provide a new opportunity in future disease treatment.

## 1 Introduction

Despite extensive efforts worldwide, cancer remains one of the leading causes of mortality (Sung et al., 2021). Surgery or radiotherapy is an effective strategy for the treatment of local tumors. In the remaining cases, chemotherapy is the main priority in cancer treatment (Chu and Sartorelli, 2018). However, chemotherapy is always accompanied by severe side effects and low therapeutic efficacy (Zhong et al., 2021). For example, a first-line clinical drug, doxorubicin (DOX) (Hai-wen et al., 2013), has been reported to result in all kinds of toxic side effects, including cardiotoxicity and alopecia, due to its low uptake by cancer cells (Zhao et al., 2019). Therefore, developing a new technology to enhance drug utilization is extremely urgent in order to reduce the side effects and improve efficacy.

Considering the physiological roles of bioelectrical signals in cell biology, electrical stimulation (ES) was introduced to improve some operations in many biological scenes. For example, a well-known microbiology technique called electroporation takes advantage of an electrical field to change the permeability of the cell membrane, allowing biomolecule transportation or even cell fusion (Zhang et al., 2018). Recently, ES has been demonstrated to have the potential to be a promising strategy for drug delivery and therapy. Karan Gulati’s group combining electrical drug delivery and electrical stimulation therapy achieved beneficial results in bone implantation (Gulati et al., 2016). Cassandra L. Weaver’s group developed a drug delivery nanocomposite controlled by electricity to meet various therapeutic needs (Weaver et al., 2014). With the concerted efforts of Pei-Chi Lee and his colleagues, increased nanomedicine accumulation within tumor tissue stimulated by electrical single-walled carbon nanotubes helps to delay tumor growth (Lee et al., 2016). However, most of these techniques require an external power source, greatly increasing the cost and inconvenience, thereby limiting their further application.

Triboelectric nanogenerators (TENGs) (Fan et al., 2012a; Wang et al., 2014a; Zheng et al., 2014; Cheng et al., 2018; Qin et al., 2020; Zhang et al., 2020; Zhang et al., 2021a), which were invented to harvest unordered ambient mechanical energy on the basis of triboelectric effect and maxwell’s displacement current (Wang, 2017), have been proved to be a simple, low-cost, and portable power source for self-powered devices and systems (Wang, 2013; Zheng et al., 2016; Cheng et al., 2017; Wang et al., 2018; Hao et al., 2021; Shang et al., 2021; Wang et al., 2021). A series of methods were investigated to increase the output performance of TENGs, such as material selection (Wang, 2013), surface roughness increment (Fan et al., 2012b; Zhang et al., 2015), surface charge injection (Wang et al., 2014a; Wang et al., 2014b), charge pump method (Cheng et al., 2018), step-down circuit (Qin et al., 2018), and multiple channel method (Zhang et al., 2022a). As a result, TENG’s output voltage and peak current have been increased to more than ten thousand volts (Li et al., 2021) and hundreds of milliamperes (Cheng et al., 2013), and its output charge density is up to millicoulombs per square meter (Zhang et al., 2022a). As the output has been ameliorated to a very high level, TENGs are widely applied to establish self-powered nanodevices or nanosystems. For example, TENG’s output changed under different driving forces, making it available to sense and detect these applied forces (Chen et al., 2022); through connecting TENGs together with sensors, UV light intensity, gas concentration, and other environmental information could be detected to realize the application of self-powered detection (Zheng et al., 2014; Su et al., 2018; Zhao et al., 2018; Zheng et al., 2022); using the current generated by TENGs, self-powered anticorrosion was realized (Wang et al., 2014a). Furthermore, through the use of display units or wireless transmission technology, the sensing result could be displayed directly or sent out for further processing (Zheng et al., 2014; Cheng et al., 2017; Zhang et al., 2022b). Using TENGs as a power source, many medical applications have been developed (Zhang et al., 2021b), and several self-powered systems have been designed for drug delivery (Wang et al., 2016; Bok et al., 2018; Liu et al., 2019). Among these systems, nanoneedles are considered one of the most effective methods for drug delivery, but bring up two problems: first, the active electrical area is usually very small, which makes it difficult to stimulate enough cells for therapy; second, fabrication of the nanoneedles and extra devices is always complicated and expensive. Thus, a simple and self-powered delivery system is required for current medicines.

In this work, we designed a very simple self-powered drug delivery system in order to reduce the severe side effects and enhance the therapeutic efficacy of chemotherapy. In this system, a disk TENG (D-TENG) is designed to provide a stable current, which performs the electrical stimulation towards cells/tissues within two Au electrodes. An *in vitro* experiment showed that using this self-powered drug delivery system, the utilization efficiency of Dox was effectively increased.

## 2 Materials and Methods

### 2.1 Materials

DOX was provided by Aladdin (Hangzhou, China). Mouse breast cancer cells (4T1 cell line) were purchased form the American Type Culture Collection (ATCC). PVDF powder was provided by Alfa Aesar (Shanghai, China). PA powder was provided by Macklin (Shanghai, China). All organic solvents were provided by Sinopharm Chemical Reagent (Shanghai, China).

### 2.2 Preparation of PA and PVDF Solutions (Weaver et al., 2014)

To prepare the PA solution, 2 g of PA powder was added to 16 g of formic acid and then stirred for 30 min to make the PA powder dissolve. To prepare the PVDF solution, 3.75 g of PVDF powder was added to 8.5 g of N,N-dimethylacetamide (DMAC) and 12.75 g of acetone and then stirred at 60°C for 30 min to make the powder dissolve.

### 2.3 Fabrication of the D-TENG

**FIGURE 1:**
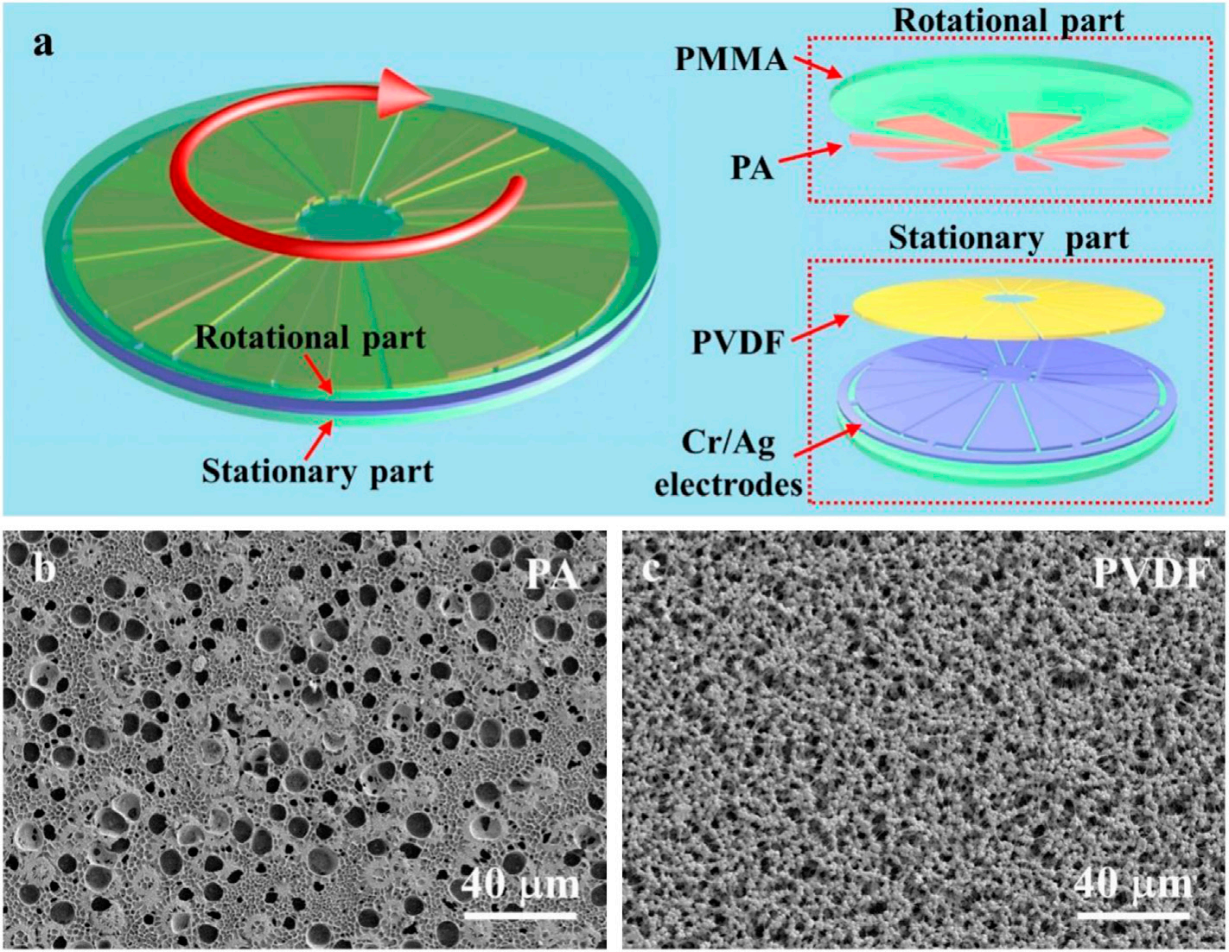
Design of the D-TENG. **(A)** Schematic diagram of the D-TENG. **(B)** Scanning electron microscope (SEM) image of the spin-coated PA film. **(C)** SEM image of the spin-coated PVDF film.

Two pieces of PMMA sheet were first cleaned in sequence with ethanol and deionized water. The PA solution was spin-coated on one of the PMMA sheets at a speed of 1500 rpm for 60 s and then left for 30 min to make sure the solution is dry. The sheet was cut into the needed shape with 3 cm of radius to make the rotational part of the D-TENG. On the other piece of the PMMA sheet, Cr and Ag films were sputtered on one surface to make the Cr/Ag electrode, and PVDF solution was spin-coated on the Cr/Ag film at the speed of 1500 rpm for 60 s and then left for 30 min to make sure the solution is dry. The sheet was then cut into the needed shape; in this process, the Cr/Ag electrode was cut into two pieces, as shown in Figure 1A and two copper wires were connected to the two electrodes to make the stationary part of the D-TENG. At last, two parts of the D-TENG were assembled with the PA film in contact with the PVDF film and the center of the sheets aligned.

### 2.4 Fabrication of the Tailor-Made Culture Dish With Au Electrodes

Au electrodes with the needed shape were sputtered on the inner side of the bottom of an ordinary culture dish. Then, two copper wires were connected to the Au electrodes at the edge of the dish to make sure only the Au electrodes will contact the culture solution. The prepared culture dish with Au electrodes was sterilized with medicinal alcohol and UV light before cell culture.

### 2.5 Flow Cytometric Analysis of Drug Utilization Efficiency

4T1 cells were seeded in plates (3 × 10^5^ cells/well) and cultured overnight. On the second day, media was changed and replaced with fresh media containing DOX. Cells were treated with/without TENG current stimulus for 1 h. The cells treated without DOX or ES were used for the control. 4T1 cells were cultured for another 24 h. Then, the cells were harvested and analyzed by a flow cytometer (BD Biosciences).

## 3 Results and Discussion

As Figure 1A shows, the D-TENG comprises a rotational part and a stationary part. The rotational part contains a PMMA sheet as the substrate and nine PA films which act as a kind of friction layer and are attached to the PMMA sheet. The PA films are cut into circular sectors and ring-distributed on the PMMA sheet with sectorial spaces of the same size. The stationary part contains a PMMA sheet as the substrate, two Cr/Ag electrodes and the sectorial PVDF films which act as the other kinds of friction layers attached to the electrodes. Each electrode contains nine sectorial units with the same size and distribution as the PA films. So, when the D-TENG is rotated, the PA films overlap with two electrodes alternately. To clearly observe the materials coating in the D-TENG, PA films (Figure 1B) and PVDF films (Figure 1C) were characterized using the scanning electron microscope (SEM). As the picture shows, rough surfaces with holes or particles formed naturally in the fabrication process of the films will help to increase the performance of the D-TENG. Details of the fabrication process are shown in the methods.

**FIGURE 2:**
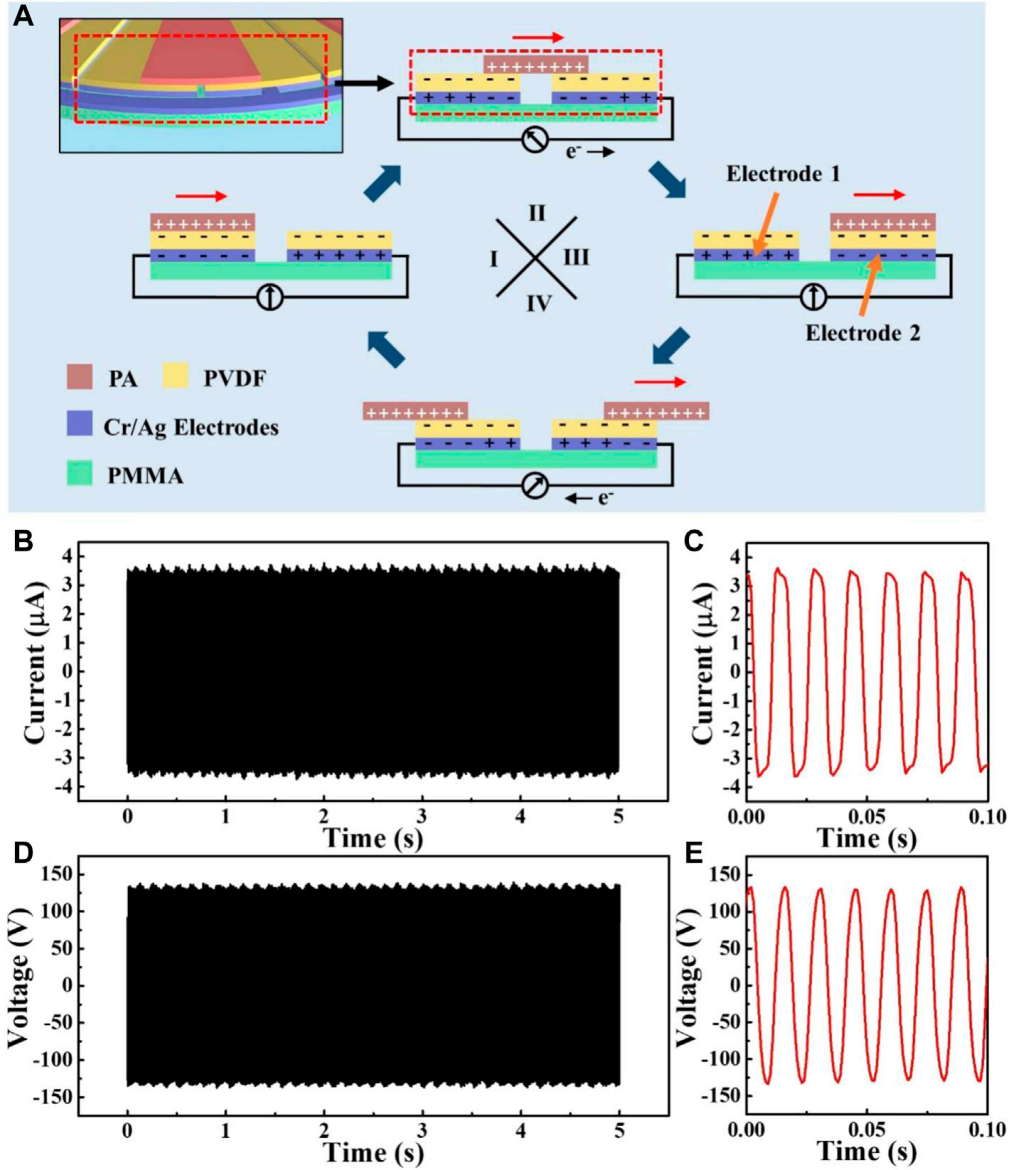
Output of the D-TENG. **(A)** Schematic diagram of the working mechanism of the D-TENG. **(B)** Output current of the D-TENG. **(C)** Enlarged view of D-TENG’s output current. **(D)** Output voltage of the D-TENG. **(E)** Enlarged view of D-TENG’s output voltage.

Figure 2A shows the working mechanism of the D-TENG. When the rotational part is rotated, PA films slide across the PVDF films. In this process, PA films rub with PVDF films. As a result, PA films take positive charges and PVDF films take negative charges. The total area of the PVDF films is twice that of the PA films’, and charge density of the PA films is twice of the PVDF films’. When PA films overlap with electrode 1 (state I), though the positive charge on the PA film is partly offset by the negative charge, the residual charge will induce a negative charge in electrode 1. At electrode 2, the negative charge taken by the PVDF films on its surface will induce a positive charge in it. As a result, electrode 1 will take a negative charge and electrode 2 will take a positive charge. When PA films slide from electrode 1 to electrode 2 (state II), the overlapping area of electrode 1 with the PA films decreases, the overlapping area of electrode 2 increase, and electrons move from electrode 1 to electrode 2 through the external circuit and generate current in the circuit. When the PA films overlap with electrode 2 (state III), electrode 1 takes a positive charge and electrode 2 takes a negative charge. And when PA films slide from electrode 2 to electrode 1 (state 4), electrons move from electrode 2 to electrode 1 and generate reversed current in the circuit. So, following the D-TENG work, one round from states I to IV will continue to the next round, which periodically generates alternating current in the circuit. As there are nine sectorial PA films in the device, each revolution of the D-TENG will generate nine forward and nine reversed current peaks in the circuit.

The D-TENG’s output was then measured with a rotational frequency of about 7.4 Hz. Its output current was about 3.7 μA (Figures 2B,C), and its output voltage was about 135 V (Figures 2D,E). The frequency of the output current and voltage was about 67 Hz, which was about nine times the D-TENG’s driving frequency. The integral results (Supplementary Figure S1) showed that the transferred charge of each peak was about 21 nC, and the D-TENG could export 2.8 μC in one second. This indicated the average output current of the D-TENG was about 2.8 μA, which achieved the comparable level commonly used in cell electrical stimulation (Macfelda et al., 2017; Yuan et al., 2021).

**FIGURE 3:**
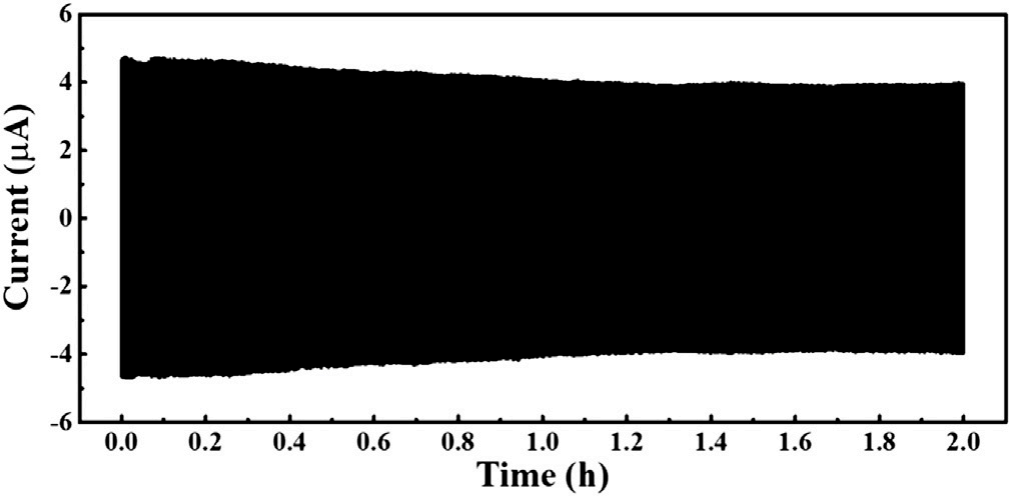
Output current of the D-TENG working continuously for 2 h.

As a source of energy, the stability of the D-TENG is one of the most important properties. Figure 3 shows the output current of the D-TENG working continuously for 2 hours. As expected, D-TENG’s output decreased slightly in the first 1 hour, until its output current stabilized at about 4 μA. This good stability and intensity indicate the D-TENG has its own superiority for self-powered drug delivery.

Using this D-TENG as a power source, we designed a simple self-powered electrical stimulation system. The system contains the D-TENG, a rectifier bridge to convert the D-TENG’s alternate current to direct current, and two Au electrodes to deliver the current. The Au electrode is used to prevent the possible electrochemical reaction. The cells/tissues stimulated by the current will increase the drug uptake efficiency.

**FIGURE 4:**
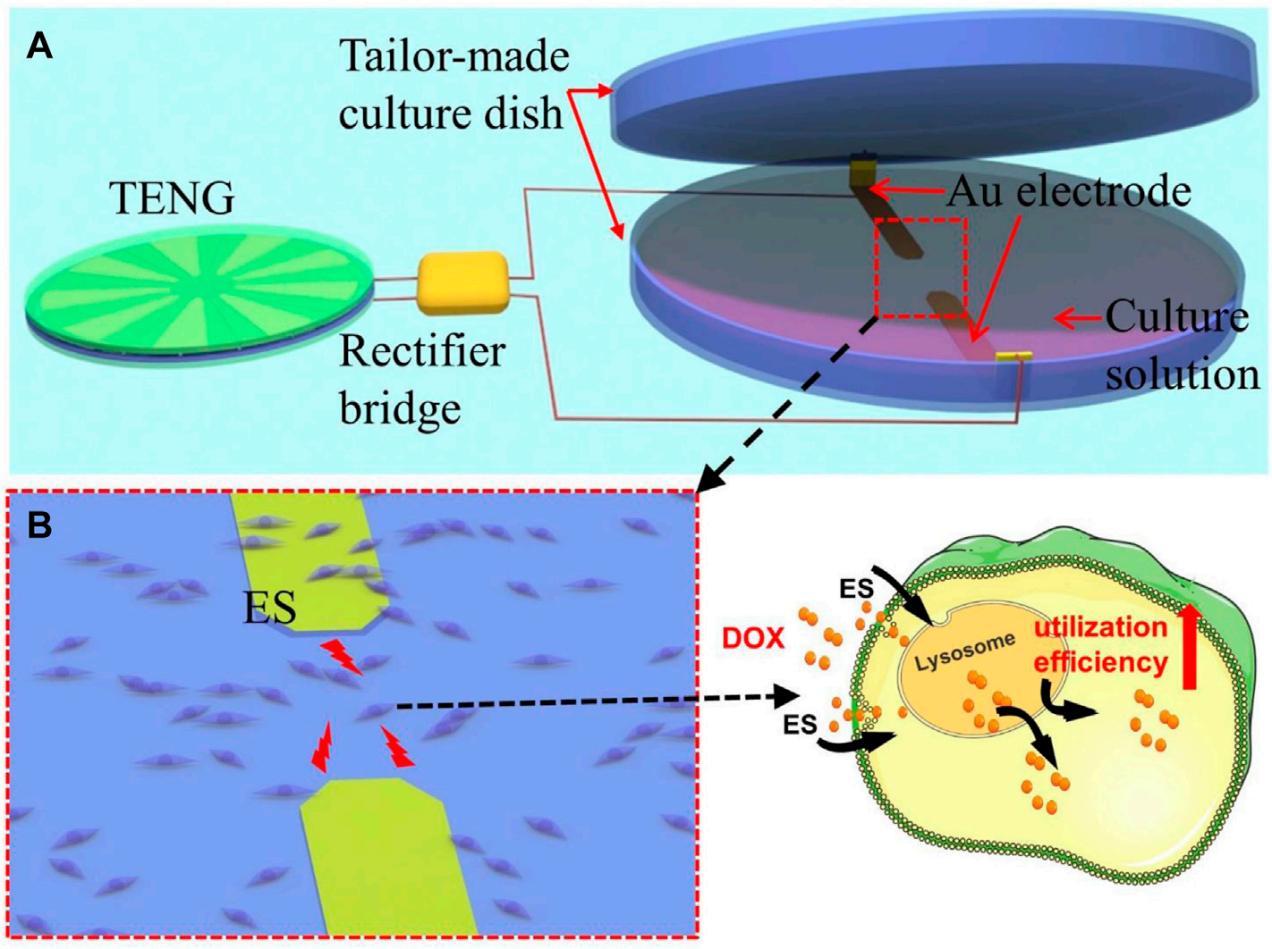
*In vitro* self-powered drug delivery equipment. **(A)** Schematic diagram of the equipment. **(B)** Schematic diagram of the cells and Dox cocultured after electrical stimulation.

An *in vitro* self-powered drug delivery experiment was then performed to check the effect of drug delivery using the simple drug delivery system. Figure 4A shows the equipment used in the *in vitro* experiment. In this equipment, the Au electrodes were deposited on the inner side of an ordinary culture disk (which consists of the tailor-made culture dish marked in the picture), which was used to culture and simulate the cells. Once the D-TENG is working, cells grow in the area between the two electrodes, which will be simulated. Figure 4B shows the mouse breast cancer cells (4T1 cell line) and Dox were cocultured in self-powered electrical stimulation system; after electrical stimulation, the uptake efficiency of cells for Dox will be determined.

**FIGURE 5:**
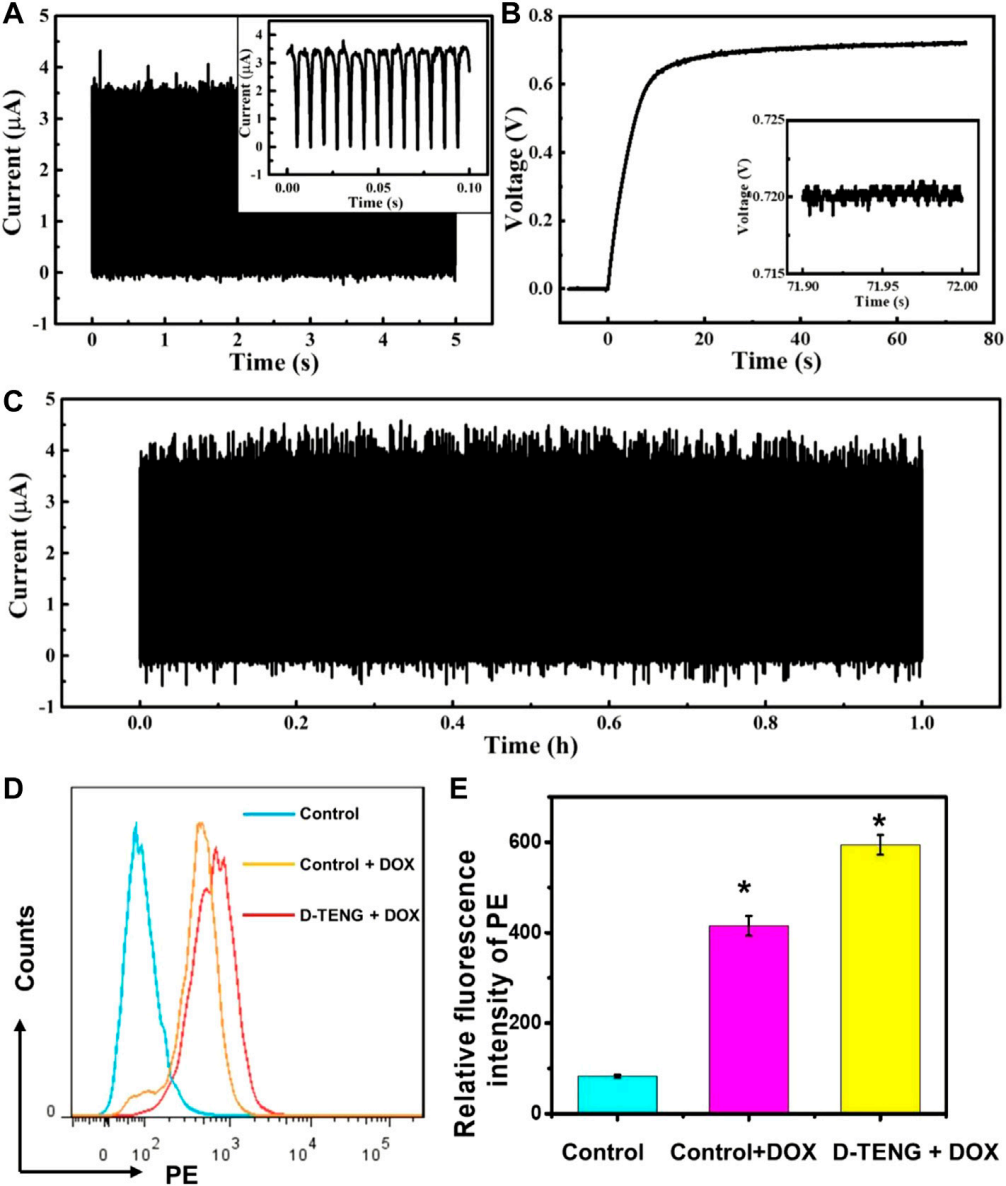
*In vitro* enhancing drug utilization efficiency. **(A)** The current transmitted to the tailor-made culture dish. **(B)** The voltage between the Au electrodes. **(C)** The current is transmitted to the tailor-made culture dish in the simulation process. **(D)**. The uptake efficiency of 4T1 cells for Dox under 1 h stimulation was analyzed 24 h after stimulation. **(E)** Quantitative analysis of **(D)**. Unpaired *t* test. *n* = 3, *p < 0.05, ns represents no significant difference.

In this experiment, mouse breast cancer cells (4T1 cell line) were seeded in culture plates. These cells were divided into three groups, including control, control with DOX (with no current stimulus), and D-TENG with DOX groups. In D-TENG with DOX groups, during the D-TENG 1 h continuous stimulation, the current transmitted to the tailor-made culture dish and the voltage between the Au electrodes were measured (Figures 5A,B). The D-TENG’s output current was converted to direct current with a peak current of 3.5 μA and an average current of 2.9 μA (shown in Supplementary Figure S2). The voltage between the Au electrodes increases rapidly in the first 10 s and stabilizes at 0.72 V. Figure 5C shows that the current was stable during the stimulation process. Afterwards, 4T1 cells were cultured for another 24 h, and the cells were harvested and analyzed by flow cytometer (BD Biosciences). The flow cytometric results indicated that this self-powered drug delivery system could significantly improve the utilization efficiency of Dox (Figures 5D,E). Therefore, the electrical stimulation of D-TENG might be used to improve drug utilization efficiency for disease treatment in the future.

In conclusion, we designed a very simple self-powered drug delivery system that contains a D-TENG, a rectifier bridge, and two Au electrodes. At a working frequency of about 7.4 Hz, the D-TENG generates a peak current of 3.7 μA and an average output current of 2.8 μA. Considering the wide ranges were applied in previous works, the output of the D-TENG is powerful enough to stimulate the cells. Also, the D-TENG has good performance to provide stable current for a long time. The output current of the D-TENG is converted to direct current by the rectifier bridge to stimulate the cells/tissues between the Au electrode. An *in vitro* experiment showed this self-powered drug delivery system could significantly improve the utilization efficiency of Dox. The next step is to establish a biocompatible system to meet the wearable or transplantable needs in clinical trials. Taken together, we developed a stable and powerful D-TENG system for drug delivery, which will be part of chemotherapy in future treatment.

## Data Availability

The raw data supporting the conclusions of this article will be made available by the authors without undue reservation.

## Supplementary Material

Supplementary Figure S1 The integral results of the D-TENG’s output current. (a) The integral result of the D-TENG’s output current without any additional process, which shows the output charge pure peak. (b) The integral result of the absolute values of D-TENG’s output current, which shows the total output charge.

Supplementary Figure S2 The integral results of the current transmitted to the tailor-made culture dish, which shows the total charge flowing through the culture solution.
