# Supplementary material for: Enhancing Drug Utilization Efficiency via Dish-Structured Triboelectric Nanogenerator
Source: Front Bioeng Biotechnol. 2022 Jul 6;10:950146. doi: 10.3389/fbioe.2022.950146 (PMC9298755; doi:10.3389/fbioe.2022.950146)
Supplement: Supplementary file 1 [file DataSheet1.pdf]

Supplementary Materials

Enhancing Drug Utilization Efficiency via Dish-structured Triboelectric Nanogenerator

Qu Chen[1,2], Wenjing Deng[6], Jingjin He[2], Li Cheng[3*], Pei-Gen Ren[4,5*], Yang Xu[6*]

[1]Institute of Biomedicine and Biotechnology, Shenzhen Institute of Advanced Technology, Chinese Academy of Sciences, Shenzhen, 518055, China.

[2]Shenzhen International Institute for Biomedical Research, Shenzhen, 518110, China.

[3]School of Materials and Energy, Lanzhou University, Lanzhou 730000, China.

[4]Center for Energy Metabolism and Reproduction, Shenzhen Institute of Advanced Technology, Chinese Academy of Sciences, Shenzhen, 518055, China.

[5]Shenzhen College of Advanced Technology, University of Chinese Academy of Sciences, Shenzhen, 518055, China.

[6]School of Basic Medical Sciences, Southern Medical University, Guangzhou 510515 Guangdong, China.

***Correspondence:**

Li Cheng Email: chengl2007@live.cn

Pei-Gen Ren Email: pg.ren@siat.ac.cn

Yang Xu Email: yangxu@ucsd.edu

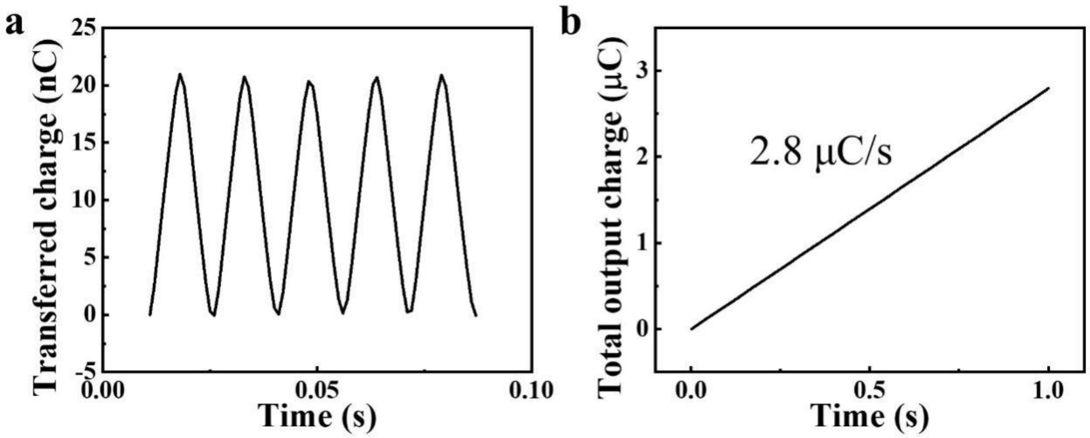

Figure S1. The integral results of the D-TENG's output current. (a) The integral result of the D-TENG's output current without any additional process, which shows the output charge pure peak. (b) The integral result of the absolute values of D-TENG's output current, which shows the total output charge.

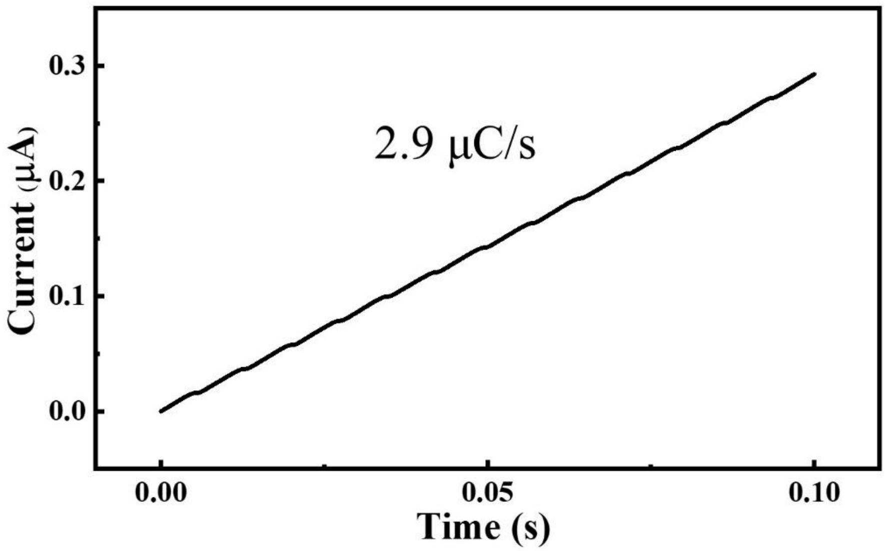

Figure S2. The integral results of the current transmitted to the tailor-made culture dish, which shows the total charge flowing through the culture solution.
